# Self-Rated Health and Social Exclusion: Does Gardening Moderate This Relation? Evidence from the German Ageing Survey

**DOI:** 10.3390/ijerph16101834

**Published:** 2019-05-23

**Authors:** André Hajek, Hans-Helmut König

**Affiliations:** Department of Health Economics and Health Services Research, Hamburg Center for Health Economics, University Medical Center Hamburg-Eppendorf, 20246 Hamburg, Germany; h.koenig@uke.de

**Keywords:** social exclusion, social isolation, subjective health, self-rated health, gardening

## Abstract

The aim of the present study was to examine whether the association between self-rated health and social exclusion can be moderated by the frequency of gardening in the total sample and stratified by sex. Cross-sectional data employed in this study came from the fifth wave of the German Ageing Survey (*n* = 5048), a nationally representative sample comprising non-institutionalized individuals aged 40 and above. A single-item measure was used to quantify self-rated health (ranging from 1 = very good to 5 = very bad). An established scale developed by Bude and Lantermann was used to assess social exclusion. Moreover, individuals reported the frequency of work in the garden (daily; several times a week; once a week; 1-3 times a month; less often; never). Poorer self-rated health was associated with feelings of social exclusion. The frequency of gardening significantly moderated the association between these factors in women. This cross-sectional study emphasizes the moderating role of gardening in the relation between self-rated health and social exclusion in women. Longitudinal studies are required to validate the present findings.

## 1. Introduction

Self-rated health usually refers to the absence of ill-health (and is also associated with life situation, fitness, health behavior and personal experiences). Poor self-rated health is not only associated with subsequent morbidity or mortality [1,2], but also with future social exclusion (social isolation and social exclusion are used interchangeably throughout the manuscript) [3]. The association between poor self-rated health and social exclusion might be explained by the fact that individuals suffering from bad self-rated health might feel unable to engage in typical activities of daily living (e.g., leisure activities performed outdoor) [4]. In addition, those individuals might compare with other individuals who are better off (in terms of health). The (i) inability to engage in activities of daily living and (ii) negative health comparisons might lead to negative emotions such as frustration or anger or to feelings that one does not belong to the society. Ultimately, these factors might lead to feelings of social exclusion [4,5]. With advanced age, it becomes more likely that individuals become more socially excluded for various reasons (e.g., functional impairment, or loss of the spouse), highlighting the importance of social exclusion in late life [6]. 

In a recent study, it has been shown that the use of mobile phone and the internet can alleviate the relation between self-rated health and social exclusion [3]. It also appears plausible to us that other factors can moderate this association. We assume that gardening might be one of these factors (e.g., act of planting of plants or trees as well as watering plants or activities that are more complex such as designing a landscape). This might have two explanations. First, it might be the case that individuals communicate with passing neighbors while performing gardening activities. This might decrease feelings of not belonging to the society. A second explanation might be that individuals perceive their plants as living beings rather than things [7]. Plants might replace or supplement other social contacts. Due to that, these individuals might feel less isolated. In sum, it appears plausible to us that gardening can alleviate the association between poor self-rated health and increased social exclusion.

Commonly, gardening is viewed as a leisure physical activity (mainly involving muscles of the legs, arms and back) in older age [8]. According to our own calculations, almost three out of four community-dwelling individuals ≥40 years have a yard or shared yard in Germany in 2014. Thus, gardening is a leisure time physical activity to which most of the older people have access (at least in Germany). Furthermore, it is generally a low-to-moderate physical activity [9], which can easily be interrupted.

As stated in a recent systematic review [10], current studies examining the consequences of gardening in late life mainly used convenience samples of individuals interested in gardening. Moreover, some studies exist based on individuals engaged in allotment gardening. In contrast to these studies, our aim was to examine whether the association between self-rated health and social exclusion can be moderated by the frequency of gardening in the total sample and stratified by sex based on a nationally representative sample of non-institutionalized individuals ≥40 years. 

## 2. Materials and Methods 

### 2.1. Sample

Provided by the Research Data Centre of the German Centre of Gerontology (DZA), the data for this study were drawn from the German Ageing Survey (DEAS). Design as well as sampling procedures have been described in detail elsewhere [11]. The DEAS study which has a cohort-sequential longitudinal design is, in brief, a population-based study of non-institutionalized individuals ≥40 years. To date, five waves (1996 to 2014) are available. New baseline samples stratified by age, gender and region were drawn in 1996, 2002, 2008, and 2014. All willing participants from former waves were re-interviewed in 2002, 2008, 2011, and 2014. In total, there were 4838 participants in 1996, 5194 participants in 2002, 8200 participants in 2008 and 4855 participants in 2011. Data collection methods were home-based interviews and self-completed questionnaires. 

In this study, data from the fifth wave (year 2014) were used because the outcome measure (social exclusion) was solely measured in this wave. Moreover, we restricted our analysis to individuals who have a yard or shared yard (using the question: How is your home equipped? Yard or use of shared yard [yes; no]) (*n* = 5048). Thus, we were only interested in comparisons between individuals with (shared) yard. To put it another way: We were not interested in individuals who did not garden because they did not have access to it. 

Written informed consent was provided by all individuals. An ethical statement for the DEAS study was not needed, as the criteria for it were not met (e.g., examination of patients, risk for the respondents, or the use of invasive methods). The DEAS study is in accordance with the Declaration of Helsinki of 1975, revised in 2013. 

### 2.2. Dependent Variable

Bude and Lantermann [12] developed a scale to quantify perceived social exclusion. This scale, consisting of four items, was used in this study. The items are: “I am worried to be left behind”, “I feel like I do not really belong to society”, “I feel that I am left out”, and “I feel excluded from society” (from 1 = “strongly agree” to 4 = “strongly disagree”). The average of at least two required valid items formed the scale (higher values correspond to higher social exclusion). In our study, Cronbach’s alpha was 0.88. The original scale had six items and the short scale used in this study had four items. Based on information from a pretest with *n* = 162, two items were removed (without the loss of reliability). In German language, these items were “Ich werde ausgegrenzt”, and “Ich habe das Gefühl, andere Menschen haben mich abgeschrieben”.

### 2.3. Independent Variables

Our independent variable of interest was self-rated health. Similar to other large cohort studies, individuals were asked to rate their current health on a 5-point Likert scale (from 1 = “very good” to 5 = “very bad”). 

In regression analysis, it was also adjusted for age, familial status (married, living together with spouse; others (married, living separated from spouse; widowed; single; divorced)), monthly net equivalent income (Organisation for Economic Co-operation and Development (OECD) scale), smoking status (non-smoker; former smoker; casual smoker; daily smoker), frequency of sports activities and alcohol consumption (categories were in both cases: ‘never’, ‘rarer than once a month’, ‘one to three times a month’, ‘once a week’, ‘several times a week’, and ‘daily’) as well as the number of physical illnesses (for example, cancer or diabetes; 0 to 11). 

In sensitivity analysis, it was also adjusted for the number of important people in regular contact (from 0 to 9) and perceived stress. The stress scale was developed by Cohen et al. [13], consisting of four items. Higher values reflect higher self-rated stress. The index score ranges from 1 to 5 Cronbach’s Alpha was 0.70 in our study. 

In another sensitivity analysis, it was also adjusted for depression (sum score ≥ 18 [14]). It was measured using the Center for Epidemiological Studies Depression Scale (15 items, 0–45). 

In further sensitivity analysis, it was additionally adjusted for the closeness of contact to the respondents’ neighbors and other neighborhood characteristics: (i) I realise what happens in the neighborhood (strongly agree; agree; disagree; strongly disagree); (ii) I talk with neighbors about what happens in the neighborhood (strongly agree; agree; disagree; strongly disagree); (iii) To a certain extent, I’m able to determine what happens in the neighborhood (strongly agree; agree; disagree; strongly disagree); (iv) How close is your contact to your neighbors? (very close, close; not really close; only rare; no contact). 

### 2.4. Moderating Variable: Frequency of Gardening

Individuals were asked ‘How often do you work in the garden during the summer months?’ in the past 12 months? (daily; several times a week; once a week; 1–3 times a month; less often; never). 

### 2.5. Statistical Analysis

Stratified by sex, sample characteristics are presented. Subsequently, multiple linear regressions were performed to examine whether the association between self-rated health and social exclusion is moderated by the frequency of gardening (including an interaction term self-rated health x the frequency of gardening). The statistical significance was determined with *p* < 0.05. Stata 15.1 was used for data analysis in this study (StataCorp, College Station, TX, USA).

## 3. Results

### 3.1. Descriptive Statistics

Descriptive statistics stratified by sex are displayed in Table 1. In total, 50.4% were male. Average age was 64.8 (±10.9) in men and 62.6 (±10.8) in women. In men, 31.1% reported daily gardening activities, and in women 33.3% reported daily gardening activities. Average social exclusion score was 1.5 (±0.5) in men and 1.6 (±0.6) in women. Further details are depicted in Table 1. 

### 3.2. Regression Analysis

Results of regression analysis are displayed in Table 2 (total sample and stratified by sex). Social exclusion was positively associated with younger age, not being married, living together with spouse (married, living separated from spouse; divorced; widowed; single), lower income, a higher number of physical illnesses and worse self-rated health in the total sample and in both sexes. 

The association between self-rated health and social exclusion was significantly moderated by gardening among women. In other words: Gardening alleviates the association between worse self-rated health and increased social exclusion. 

In sensitivity analysis (results not shown, but available upon request), the main model was extended by adding the number of important people in regular contact and perceived stress. The association was slightly lower (for example: interaction term for gardening several times a week x self-rated health was β = −0.13, *p* < 0.05 in women). However, findings remained similar. It was also adjusted for depression in another robustness check. Findings remained virtually the same (for example: interaction term for gardening once a week x self-rated health was β = −0.13, *p* < 0.05 in women). We also adjusted for the closeness of contact to the respondents’ neighbors and other neighborhood characteristics in further sensitivity analysis (please see the methods section for further details). However, the interaction terms (self-rated health x gardening) remained almost the same in terms of significance and effect size (for example: interaction term for gardening one to three times a month x self-rated health was β = −0.15, *p* < 0.05 in women). 

## 4. Discussion

The objective of this study was to investigate whether the association between self-rated health and social exclusion depends on the frequency of gardening. Regressions revealed that poorer self-rated health was associated with feelings of social exclusion and this association was significantly moderated by the frequency of gardening in women. Previous studies have shown that gardening is associated with positive health outcomes in late life [10,15], which ultimately might affect social exclusion. However, it was for example adjusted for the number of physical illnesses or depression (sensitivity analysis) in our regression model. A possible explanation for the association might be that women with bad self-rated health who are engaged in gardening activities not only have decreased levels of stress due to the physical activity [8] but also communicate with neighbors. In turn, they might feel that they belong to the society. Consequently, they might report decreased feelings of social exclusion. Another idea proposed in the introduction was that they might replace or supplement social contacts by plants. The underlying idea was that plants are recognized as living beings who can grow over time [7]. Another idea is that plants increase the feeling of contact with nature. This in turn might buffer the link between self-rated health and social exclusion. However, future research is urgently required to investigate the gardening activities in further detail (exact time, detailed description of gardening activities and relationship to the plants). 

In contrast to women, the association between self-rated health and social exclusion was not moderated by the frequency of gardening in men. While we strongly assume that gardening activities are also associated with decreased distress in men, it appears plausible to us that in contrast to women men are less involved in social activities while gardening. Moreover, they might not build a strong relationship to their plants. Furthermore, other factors (e.g., pet ownership, health comparisons) may be of greater importance in the link between self-rated health and social exclusion for men. However, further research is required to test our assumptions. 

In our study, first evidence was provided showing that the frequency of gardening can moderate the association between self-rated health and social exclusion. Data employed in this study came from a large nationally representative sample comprising non-institutionalized individuals ≥ 40 years. Social exclusion was assessed using an established scale. The frequency of gardening was measured using one item. However, gardening can vary from simple efforts of planting to complex and time-involving activities. Future research is required to investigate this factor in depth. Moreover, longitudinal studies are required to validate our findings based on cross-sectional data. Only a small sample selection bias was observed in the German Ageing Survey [11]. 

## 5. Conclusions

This cross-sectional study emphasizes the moderating role of the frequency of gardening in the relation between self-rated health and social exclusion. Identifying the relationship is of value due to the clear association between social exclusion and morbidity as well as mortality [16]. As stated by Wang and MacMillan [10], “growing plants, fruits, and vegetables of one’s choice can be a way of allowing individuals to remain connected to their family, community, and cultures”. Longitudinal studies are required to validate the present findings. 

## Figures and Tables

**Table 1:** Sample characteristics stratified by sex (*n* = 5048).

Independent Variables	Men (*n* = 2544)	Women (*n* = 2504)	*p*-Value
	N (%)/Mean (±SD)	N (%)/Mean (±SD)	
Age in years	64.8 (±10.9)	62.6 (±10.8)	<0.001
Other marital status (Single, divorced, widowed)	457 (18.0%)	732 (29.2%)	0.13
Monthly net equivalent income (€)	2130.8 (±1577.5)	1979.0 (±1387.0)	<0.001
Employment status: Employed	966 (38.0%)	1032 (41.2%)	<0.001
Retired	1463 (57.5%)	1158 (46.3%)	
Other	115 (4.5%)	314 (12.5%)	
Smoking behavior: Yes, daily	318 (12.5%)	315 (12.6%)	<0.001
Yes, sometimes	125 (4.9%)	81 (3.2%)	
No, not anymore	1145 (45.0%)	755 (30.2%)	
Never been a smoker	956 (37.6%)	1353 (54.0%)	
Frequency of alcohol consumption: Daily	486 (19.1%)	153 (6.1%)	<0.001
Several times a week	851 (33.5%)	474 (18.9%)	
Once a week	399 (15.7%)	424 (16.9%)	
One to three times a month	257 (10.1%)	374 (15.0%)	
Less frequently	362 (14.2%)	796 (31.8%)	
Never	189 (7.4%)	283 (11.3%)	
Frequency of sports activities: Daily	186 (7.3%)	208 (8.3%)	<0.001
Several times a week	686 (27.0%)	745 (29.7%)	
Once a week	423 (16.6%)	544 (21.7%)	
One to three times a month	243 (9.5%)	172 (6.9%)	
Less frequently	348 (13.7%)	262 (10.5%)	
Never	658 (25.9%)	573 (22.9%)	
Frequency of gardening: Daily	791 (31.1%)	833 (33.3%)	<0.001
Several times a week	810 (31.9%)	794 (31.7%)	
Once a week	422 (16.6%)	360 (14.4%)	
One to three times a month	207 (8.1%)	119 (4.7%)	
Less frequently	156 (6.1%)	161 (6.4%)	
Never	157 (6.2%)	237 (9.5%)	
Self-rated health (from 1 = “very good” to 5 = “very bad”)	2.5 (±0.8)	2.4 (±0.8)	0.08
Number of chronic illnesses (from 0 to 11)	2.6 (±1.8)	2.4 (±1.8)	<0.01
Social exclusion (ranging from 1 (lowest) to 4 (highest)	1.5 (±0.5)	1.6 (±0.6)	<0.01

Comparisons between the two groups were done using *t*-test and chi-square procedures.

**Table 2:** Determinants of social exclusion. Results of linear regression analysis.

Variables	Social Exclusion—Total Sample	Social Exclusion—Men	Social Exclusion—Women	Social Exclusion—Total Sample (with Interaction Term)	Social Exclusion—Men (with Interaction Term)	Social Exclusion—Women (with Interaction Term)
Age	−0.00 ***	−0.00	−0.01 ***	−0.00 ***	−0.00	−0.01 ***
	(0.00)	(0.00)	(0.00)	(0.00)	(0.00)	(0.00)
Employment: - retired (Ref.: employed)	0.07 *	0.05	0.07 +	0.06*	0.04	0.07 +
	(0.03)	(0.04)	(0.04)	(0.03)	(0.04)	(0.04)
- other (not employed)	0.14 ***	0.17 **	0.12 **	0.14 ***	0.17 **	0.11 **
	(0.03)	(0.06)	(0.04)	(0.03)	(0.06)	(0.04)
Marital status: other (married, living together with spouse/divorced/widowed/single; Ref.: married, living together with spouse)	0.08 ***	0.12 ***	0.04 +	0.08 ***	0.12 ***	0.05 +
	(0.02)	(0.03)	(0.03)	(0.02)	(0.03)	(0.03)
Monthly net equivalence income	−0.00 ***	−0.00 ***	−0.00 ***	−0.00 ***	−0.00 ***	−0.00 ***
	(0.00)	(0.00)	(0.00)	(0.00)	(0.00)	(0.00)
Smoking: - daily (Ref.: never been smoker)	0.01	0.00	−0.00	0.01	0.01	0.00
	(0.03)	(0.03)	(0.04)	(0.03)	(0.03)	(0.04)
- sometimes	−0.05	0.02	−0.15 **	−0.05	0.02	−0.15 **
	(0.04)	(0.05)	(0.06)	(0.04)	(0.05)	(0.06)
- no, not anymore	0.01	−0.01	0.01	0.01	−0.00	0.01
	(0.02)	(0.02)	(0.02)	(0.02)	(0.02)	(0.02)
Alcohol consumption: - daily (Ref.: never)	−0.13 ***	−0.15 **	−0.07	−0.13 ***	−0.16 **	−0.06
	(0.04)	(0.05)	(0.06)	(0.04)	(0.05)	(0.06)
- several times a week	−0.10 **	−0.12*	−0.07	−0.10 **	−0.13 **	−0.08
	(0.03)	(0.05)	(0.05)	(0.03)	(0.05)	(0.05)
- once a week	−0.08 *	−0.06	−0.12 *	−0.09 *	−0.07	−0.12 *
	(0.04)	(0.05)	(0.05)	(0.04)	(0.05)	(0.05)
- one to three times a month	−0.06 +	−0.06	−0.07	−0.07 +	−0.07	−0.08
	(0.04)	(0.06)	(0.05)	(0.04)	(0.06)	(0.05)
- less frequently	−0.02	−0.01	−0.04	−0.03	−0.02	−0.03
	(0.03)	(0.05)	(0.04)	(0.03)	(0.06)	(0.04)
Frequency of sports activities: - daily (Ref.: never)	−0.01	0.01	−0.02	−0.01	0.00	−0.02
	(0.03)	(0.04)	(0.04)	(0.03)	(0.04)	(0.04)
- several times a week	−0.06 *	−0.04	−0.07 *	−0.06 **	−0.04	−0.07 *
	(0.02)	(0.03)	(0.03)	(0.02)	(0.03)	(0.03)
- once a week	−0.00	0.00	−0.01	−0.01	−0.00	−0.01
	(0.02)	(0.03)	(0.03)	(0.02)	(0.03)	(0.03)
- one to three times a month	0.00	−0.02	0.03	−0.00	−0.02	0.03
	(0.03)	(0.04)	(0.05)	(0.03)	(0.04)	(0.05)
- less frequently	−0.02	−0.03	0.01	−0.02	−0.04	0.01
	(0.03)	(0.03)	(0.04)	(0.03)	(0.03)	(0.04)
Number of physical illnesses	0.04 ***	0.04 ***	0.05 ***	0.04 ***	0.04 ***	0.05 ***
	(0.01)	(0.01)	(0.01)	(0.01)	(0.01)	(0.01)
Self-rated health (from 1 = very good to 5 = very bad)	0.10 ***	0.12 ***	0.08 ***	0.14 ***	0.08	0.19 ***
	(0.01)	(0.02)	(0.02)	(0.03)	(0.05)	(0.04)
Frequency of gardening: - daily (Ref.: never)				0.20 *	0.10	0.29 *
				(0.10)	(0.14)	(0.14)
- several times a week				0.15	−0.16	0.43 **
				(0.10)	(0.14)	(0.13)
- once a week				0.22 *	0.02	0.37 **
				(0.11)	(0.15)	(0.14)
- one to three times a month				0.05	−0.24	0.43 *
				(0.13)	(0.17)	(0.19)
- less frequently				0.08	−0.19	0.31
				(0.13)	(0.18)	(0.19)
Interaction terms: - Daily gardening (Ref. never) x self-rated health				−0.06 +	−0.01	−0.10*
				(0.04)	(0.06)	(0.05)
- Gardening several times a week x self-rated health				−0.03	0.09 +	−0.14 **
				(0.04)	(0.06)	(0.05)
- Gardening once a week x self-rated health				−0.08 +	0.01	−0.14 *
				(0.04)	(0.06)	(0.06)
- Gardening one to three times a month week x self-rated health				−0.01	0.12 +	−0.17 *
				(0.05)	(0.07)	(0.07)
- Gardening less frequently x self-rated health				−0.02	0.09	−0.11
				(0.05)	(0.07)	(0.07)
Constant	2.61 ***	2.42 ***	2.77 ***	2.45 ***	2.48 ***	2.45 ***
	(0.08)	(0.11)	(0.11)	(0.11)	(0.17)	(0.16)
Observations	5048	2544	2504	5047	2543	2504
R^2^	0.10	0.12	0.09	0.11	0.13	0.10

Comments: Beta-Coefficients are reported; robust standard errors in parentheses. *** *p* < 0.001, ** *p* < 0.01, * *p* < 0.05, + *p* < 0.10. Social exclusion was quantified using a scale developed by Bude and Lantermann [12].
